# Study the Expression of CD10 in Prostate Carcinoma and its Correlation with Various Clinicopathological Parameters

**DOI:** 10.30699/IJP.14.2.135

**Published:** 2019-06-10

**Authors:** Lalit Singh, Nisha Marwah, Namita Bhutani, Devendra Pawar, Raman Kapil, Rajeev Sen

**Affiliations:** 1 *Junior Resident, M.B.B.S, Dept. of Pathology, Pt. BD Sharma, PGIMS Rohtak, Haryana, India*; 2 *Professor, M.B.B.S, M.D, Dept. of Pathology, Pt BD Sharma, PGIMS Rohtak, Haryana, India*; 3 *Senior Resident, M.B.B.S, M.D, DNB, Dept. Of Pathology, Pt BD Shrama, PGIMS Rohtak, Haryana, India*; 4 *Professor & Head, M.B.B.S, M.S, M.Ch. Dept. of Urology, Pt. BD Sharma, PGIMS Rohtak, Haryana, India*; 5 *Professor & Head, M.B.B.S, M.D, Dept. of Pathology, Pt BD Sharma, PGIMS Rohtak, Haryana, India*

**Keywords:** CD10, Immunohistochemistry, Gleason, Grade group, Prostate carcinoma, Prostate specific antigen

## Abstract

**Background and Objective:**

Adenocarcinoma of the prostate is the second most common cause of cancer. The loss of CD10 is a common early event in human prostate cancer and is seen in lower Gleason Score malignancies while increased and altered expression is seen in high Gleason Score tumors, lymph nodes and bone metastasis.

**Material and Methods:**

This was a prospective observational study conducted on 75 patients suspected to have prostate cancer. Immunohistochemical profile was assessed for PSA, AMACR and CD10 immunostaining. The intensity of CD10 expression and pattern of CD10 staining of tumor cells was evaluated.

**Results:**

The patients were in age group of 50-90 years with a mean age of 70.97 ± 9.51 years. As the Grade Group/Gleason Score increased, the number of cases showing negative expression decreased and the pattern of expression changed from membranous to cytoplasmic to both types of expression. As the serum PSA levels increased the intensity of expression changed from focally positive to diffusely positive. The pattern of expression also changed from membranous to cytoplasmic to both (membranous + cytoplasmic) types of expression with an increase in PSA levels.

**Conclusion:**

By immunohistochemical analysis we can identify CD10 positive tumors, which may warrant more aggressive initial therapy. A number of drugs against CD10 are available based on which potential targeted therapies could be formulated.

## Introduction

Adenocarcinoma of the prostate is the second most common cause of cancer and sixth leading cause of cancer-related deaths in men worldwide. Over 95% of prostatic cancers are adenocarcinomas that arise in prostate acini (1). Clinically prostate cancer may be asymptomatic and its natural progression is relatively slow. Usually it is detected by suspicious nodule on digital rectal examination (DRE) or raised prostate specific antigen (PSA) levels. Urinary symptoms such as hesitancy, dysuria, increased frequency or hematuria occur and are seen in advanced cases (2). Suspicious findings in either DRE or serum PSA is followed by more sophisticated diagnostic techniques such as TRUS and guided biopsy (3).

Prostate specific antigen is the most important, accurate, and clinically useful biochemical marker in the prostate. The serum levels are normally less than 4 ng/ml. The elevated PSA serum levels are seen in prostatitis, infarcts, hyperplasia and transiently after biopsy, but the most important cause is prostatic adenocarcinoma. The serum levels of prostate specific antigen (PSA) are widely used to screen men for prostate cancer but the well documented lack of sensitivity and specificity has led not only to unnecessary prostate biopsies but also to the limited ability to accurately distinguish patients with or without carcinoma (4). Further research for the development and validation of more specific biomarkers for early cancer detection is warranted to help overcome limitations of PSA and improve prostate cancer detection. Numerous studies of potential serum, urine and tissue biomarkers of prostate cancer have been presented. These new biomarkers have the potential to provide an opportunity to better define groups of men at high risk of developing prostate cancer, to improve the screening techniques and to discriminate indolent versus aggressive disease (3).

Various biomarkers are used to differentiate prostatic adenocarcinoma from benign prostatic lesions such as Cytokeratins, AMACR, AGR 2, Endothelins, Cyclin D 1 and p63. Natural endopeptidase (NEP) or CD10 is a cell surface peptidase that inactivates neuropeptide growth factor implicated in prostate cancer progression. It plays an important role in the pathogenesis of prostate cancer. The loss of CD10 is a common early event in human prostate cancer and is seen in lower Gleason Score malignancies while increased and altered expression is seen in high Gleason Score tumors, lymph nodes and bone metastasis (3). 

## Materials and Methods

This was a prospective observational study conducted on 75 patients suspected to have prostate cancer on the basis of clinical features, radiological imaging and confirmation on prostatic biopsy. All types of prostatic specimens including transuretheral resection of prostate (TURP), needle biopsy, TRUS guided biopsy and prostatectomy having carcinoma were included. Inadequate biopsies and poorly preserved prostatic specimens were excluded from the study. All the prostatic specimens were subjected to the careful and detailed gross examination. The accurate weight, size and color of TURP chips were noted. All the TURP chips were taken until four cassettes were filled. Haematoxylin and eosin (H & E) staining was carried out for routine paraffin sections as per the standard procedure. Immunohistochemical staining was performed using standard technique mounting of 3-4 µm sections on the slides coated with suitable tissue adhesive. This was followed by deparaffinization of sections in xylene and rehydration through graded alcohols. Antigen retrieval was done using fully automated system- Dako PT Link. This system requires pre-treatment with heat induced epitope retrieval. The application of optimally diluted primary antibody for 60 minutes was done. Immunohistochemical profile was assessed by subjecting one section from the representative block to the PSA, AMACR and CD10 immunostain. The intensity of CD10 expression and pattern of CD10 staining of tumor cells was evaluated and categorized as:

**-Intensity of staining:**

0: Negative 

1: focally positive

2: diffusely positive

**-Pattern of CD10 expression:**

Membranous

Cytoplasmic

**-For statistical analysis**

- Staining in <5% of tumor cells was considered as negative.
- Staining in 5-20% of tumor cells – focally positive.
- Staining in >20% of tumor cells – diffusely positive.

A descriptive study was carried out for all variables included in the study. The whole data was entered in Microsoft excel master sheet and analyzed using SPSS, v20 software. The obtained results were interpreted and descriptive statistics (mean, standard deviation, range, percentage) were applied wherever appropriate. Where the data was qualitative, chi square test was used to assess the association between those parameters. A value of *P*<0.05 was taken as significant whereas *P*>0.05 was taken as insignificant.

## Results

The histopathological diagnosis was established on routine H & E staining. The histological grading was done by WHO Grade Group system based on Gleason Score. The patients were in age group of 50-90 years with a mean age of 70.97 ± 9.51 years. The cases were categorized according to Gleason Score and WHO Grade Group system as follows:

1. Grade Group I (Gleason Score ≤ 6) – Only individual discrete well-formed glands.
2. Grade Group II (Gleason Score 3+4=7) – Predominantly well-formed glands with a lesser component of poorly-formed/fused/cribriform glands.
3. Grade Group III (Gleason Score 4+3=7) – Predominantly poorly-formed/fused/cribriform glands with lesser component of well-formed glands.
4. Grade Group IV (Gleason Score 8)

- Only poorly-formed/fused/cribriform glands or
- Predominantly well-formed glands and lesser component lacking glands or
- Predominantly lacking glands and lesser component of well-formed glands.

1. Grade Group V (Gleason Score 9-10) – Lack of gland formation (or with necrosis) with or without poorly-formed/fused/cribriform glands (5).

**Figure 1:**
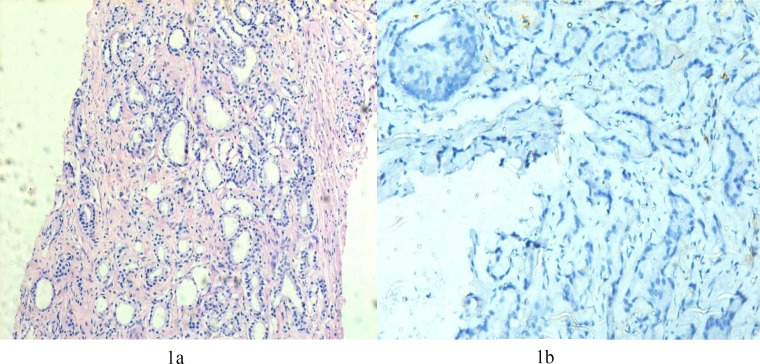
a: Photomicrograph depicting adenocarcinoma Gleason Score (3+3=6) (H&E, 100X) b: On IHC, CD10 negative (200X)

**Figure 2:**
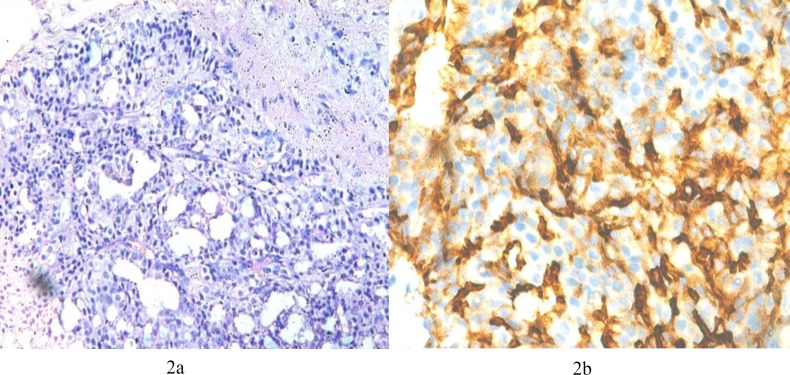
a: Photomicrograph showing Gleason Score 4+3=7 (H & E, 200X)

**Figure 3:**
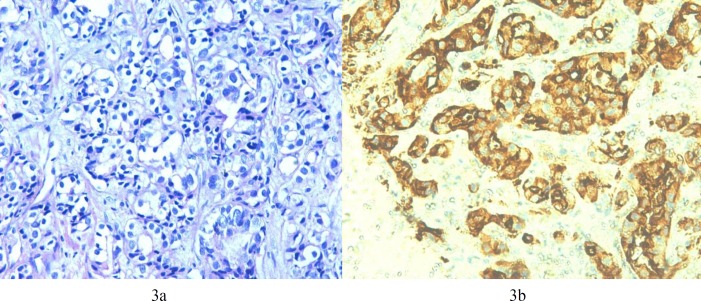
a: On H & E, Gleason Score 4+4=8, WHO Grade Group IV (200X)

Cases having Gleason Score ≥ 6 were considered as malignant. We had a total of 12% of cases with Gleason Score 6 (Figures 1a and 1b), 40% of cases had Gleason Score 7 (Figures 2a and 2b) and 20% of cases with Gleason Score 8 (Figures 3a and 3b). A total of 24% of cases had Gleason Score 9 while only 4% were categorized having Gleason Score 10. Various correlations were made between different variables. 

The majority of cases i.e 88.9% with Gleason Score 6 (WHO Grade Group I) were negative for the CD10 expression (Figures 1a and 1b). Half of the cases (50%) with Grade Group II were negative and the remaining half showed focal positivity (Figures 2a and 2b). As the Grade Group/Gleason Score increased, the number of cases showing negative expression decreased from 27.8% in Grade Group III to 6.7% in Grade Group IV while none of the case of Grade Group V showed negative expression for CD10. Also with increase in Gleason Score, the intensity of expression changed from focally positive to diffusely positive (*P*<0.001). A total of 38.9% of cases of Grade Group III, 66.7% of Grade Group IV and 95.2% of cases of Grade Group V were diffusely positive for the expression (Tables 1 and 2).

As the Gleason Score/Grade Group increased the pattern of expression changed from membranous to cytoplasmic to both types of expression. None of the cases of Grade Group I and II showed cytoplasmic positivity. The 11.1% of cases of Grade Group III, 20% of cases of Grade Group IV and 42.9% of cases of Grade Group V showed cytoplasmic expression (*P*<0.001) (Table 3).

b: On IHC, CD10 showing membranous positivity (200X)

**Table 1:** Correlation between Gleason Score and pattern of CD10 expression

Gleason Score	CD10 expression pattern				Total
	Negative	Membranous	Cytoplasmic	Both	
6	8 (88.9%)	1 (11.1%)	0 (0.0%)	0 (0.0%)	9 (100.0%)
7	11 (36.7%)	16 (53.3%)	2 (6.7%)	1 (3.3%)	30(100.0%)
8	1 (6.7%)	3 (20%)	3 (20%)	8 (53.3%)	15 (100.0%)
9	0 (0.0%)	1 (5.6%)	8 (44.4%)	9 (50.0%)	18 (100.0%)
10	0 (0.0%)	0 (0.0%)	1 (33.3%)	2 (66.7%)	3 (100.0%)
TOTAL	20 (26.7%)	21 (28.0%)	14 (18.7%)	(26.7%)	75(100.0%)

**Table 2:** Correlation between WHO Grade Group and intensity of CD10 expression

WHO Grade Group	CD10 expression intensity			Total
	Negative	Focally positive	Diffusely positive	
I	8 (88.9%)	0 (0.0%)	1 (11.1%)	9 (100.0%)
II	6 (50.0%)	6 (50.0%)	0 (0.0%)	12 (100.0%)
III	5 (27.8%)	6 (33.3%)	7 (38.9%)	18 (100.0%)
IV	1 (6.7%)	4 (26.7%)	10 (66.7%)	15 (100.0%)
V	0 (0.0%)	1 (4.8%)	20 (95.2%)	21 (100.0%)
TOTAL	20 (26.7%)	17 (22.7%)	38 (50.7%)	75 (100.0%)

**Table 3:** Correlation between WHO Grade Group and pattern of CD10 expression

WHO Grade Group	CD10 expression pattern				Total
	Negative	Membranous	Cytoplasmic	Both	
I	8 (88.9%)	1 (11.1%)	0 (0.0%)	0 (0.0%)	9(100.0%)
II	6 (50.0%)	6 (50.0%)	0 (0.0%)	0 (0.0%)	12 (100.0%)
III	5 (27.8%)	10 (55.6%)	2 (11.1%)	1 (5.56%)	18 (100.0%)
IV	1 (6.7%)	3 (20.0%)	3 (20.0%)	8 (53.3%)	15 (100.0%)
V	0 (0.0%)	1 (4.8%)	9 (42.9%)	11 (52.4%)	21 (100.0%)
TOTAL	20 (26.7%)	21 (28.0%)	14 (18.7%)	20 (26.7%)	75 (100.0%)

According to the serum PSA levels cases were divided into three groups <10, 11-20 and >20 ng/ml. As the serum PSA levels increased, the intensity of expression changed from focally positive to diffusely positive. A total of 56.1% of cases having serum PSA >20 ng/ml showed diffuse positivity while 21.2% of cases were focally positive. None of the cases having PSA 11-20 ng/ml showed diffuse CD10 expression and 28.6% of the cases were focally positive (Tables 4 and 5). The pattern of expression also changed from membranous to cytoplasmic to both (membranous + cytoplasmic) types of expression with increase in PSA levels (Figures 3a and 3b). However, no significant correlation was noted between serum PSA levels and WHO Grade Group (Table 6).

b: On IHC CD10 showing both membranous as well as cytoplasmic positivity (200X)

**Table 4:** Correlation between serum PSA levels and pattern of CD10 expression

PSA Levels (ng/ml)	CD10 expression pattern				Total
	Negative	Membranous	Cytoplasmic	Both	
<10	0 (0.0%)	2 (100.0%)	0 (0.0%)	0 (0.0%)	2 (100.0%)
11-20	5 (71.4%)	1 (14.3%)	1 (14.3%)	0 (0.0%)	7 (100.0%)
>20	15 (22.7%)	18 (27.3%)	13 (19.7%)	20 (30.3%)	66 (100.0%)
TOTAL	20 (26.7%)	21 (28.0%)	14 (18.7%)	20 (26.7%)	75 (100.0%)

**Table 5:** Correlation between Gleason Score and serum PSA levels

Gleason Score	PSA levels (ng/ml)			TOTAL
	<10	11-20	>20	
6	0 (0.0%)	2 (22.2%)	7(77.8%)	9 (100.0%)
7	2 (6.7%)	3 (10.0%)	25 (83.3%)	30 (100.0%)
8	0 (0.0%)	2 (13.3%)	13 (86.7%)	15 (100.0%)
9	0 (0.0%)	0 (0.0%)	18 (100.0%)	18 (100.0%)
10	0 (0.0%)	0 (0.0%)	3 (100.0%)	3 (100.0%)
Total	2 (2.7%)	7( 9.3%)	66 (88.0%)	75 (100.0%)

**Table 6:** Correlation between WHO Grade Group and serum PSA levels

WHO Grade Group	PSA LEVELS(ng/ml)			TOTAL
	<10	11-20	>20	
I	0 (0.0%)	2 (22.2%)	7 (77.8%)	9 (100.0%)
II	1 (8.3%)	0 (0.0%)	11 (91.7%)	12 (100.0%)
III	1 (5.6%)	3 (16.7%)	14 (77.8%)	18 (100.0%)
IV	0 (0.0%)	2 (13.3%)	13 (86.7%)	15 (100.0%)
V	0 (0.0%)	0 (0.0%)	21 (100.0%)	21 (100.0%)
TOTAL	2 (2.7%)	7 (9.3%)	66 (88.0%)	75 (100.0%)

## Discussion

Prostate cancer is a heterogeneous disease with different clinical presentations, response to therapy and long term outcomes. There are several challenges in treating the disease; the most difficult involves identifying and distinguishing aggressive tumors from those that remains indolent with little detriment to the patient. Current emerging biomarkers aim to enable the determination of an appropriate treatment strategy for the individual patients to detect advanced disease at an earlier stage, and to predict metastatic cancer and re-occurring disease following prostatectomy. Many studies (6-12) have demonstrated a role of both neuropeptides and CD10 in pathogenesis, progression, angiogenesis and metastatic potential of prostatic adenocarcinoma. 

In our study, we investigated the localization and intensity of expression of CD10 in the cells of prostatic carcinoma. The CD10 expression was seen in 73.4% of the cases of carcinoma prostate. The cases with low Gleason Score of 6 and 7 were negative or focally positive while high score tumors of 9 and 10 with Grade Group IV and V were diffusely positive in majority of the cases (97.2%). The frequency of CD10 expression significantly increased with high Gleason Score and Grade Group. The staining was membranous in low Gleason Score carcinomas of 6 and 7 while in high score malignancy it was cytoplasmic or both (cytoplasmic and membranous). Majority of the cases (53.3%) of Gleason Score 7 had membranous expression while 66.7% of the cases with Gleason Score 10 showed both cytoplasmic and membranous expression.

This distinct pattern of CD10 expression in relation to the histological grade was also noted in study by Tawfic S et al., Similarly, Dall Era et al (6,7) observed that in tumors with predominantly pattern 3, the percentage of positive CD10 staining was less than 5-10%. Higher percentages were found in tumors with pattern 4 or 5. Albrecht et al. (8) found that in high grade prostate tumors, the CD10 was distributed heterogeneously throughout the tissue with an extracellular, intra-cytoplasmic and partly plasma membrane bound localization. Saranya D (9) studied the expression of CD10 in 26 cases of carcinoma prostate biopsies. He analyzed that all the grade 2 components showed the absence of expression. In grade 3 tumors, 76.92% showed the absence of expression. Among grade 4 lesions, 71.43% showed intense cytoplasmic positivity while all the cases of grade 5 lesions showed diffuse cytoplasmic positivity. The results of these studies were in concordance with our study. In contrast, Vlachostergios P J et al. (10) found a significant inverse association of CD10 with Gleason Score (*P*=0.003). The comparison of correlation between Grade Groups and CD10 expression was done. The results of study conducted by Saranya D (9) were comparable to our study. The expression of CD10 in their study followed nearly the similar pattern as in our study. 

Considering serum PSA levels and CD10 expression, it was observed that most of the cases which were diffusely positive for the CD10 expression were having serum PSA >20 ng/ml (56.1%). The intensity of expression changed from negative to focal and diffuse with increase in serum PSA levels. Most of the cases with PSA levels <20 were negative for expression (55.55%). The pattern of expression also changed from membranous to cytoplasmic to both with higher PSA levels. Osman et al. (11) found no significant association of CD10 with serum PSA thus contradicting our findings. However, studies by Fleischmann A et al and Dall Era et al were in concordance with our study (12,7).

Differential expression of CD10 in various prostatic tumors has been documented in various studies. The early loss of expression by low grade tumors has been reasoned out by the specific microarray studies. The loss of CD10 expression could be due to hypermethylation of promoter region resulting into lack of synthesis of CD10 and reduced or absent expression in low grade tumors (Grade II and III) (8). The cytoplasmic localization in high grade tumors could be due to increased bound forms of CD10 with cytoplasmic heat shock proteins which drives the cell to the constant signaling pathway that is independent of growth factor signaling (7).

The major limitations of our study were incapacity to do pathological staging as majority of the cases in our study were tru-cut biopsies and majority of our patients were not followed up for PSA recurrence, free survival and lymph node metastasis. Thus, theassociation between CD10 expression and the outcome of disease could not be assessed.

## Conclusion

The differential expression of CD10 in normal and pathologically altered prostate correlates well with the known predictors of aggressive disease notably Gleason Score, Gleason Grade Group and serum PSA levels. By immunohistochemical analysis we can identify CD10 positive tumors, which may warrant more aggressive initial therapy. A number of drugs against CD10 are available based on which potential targeted therapies could be formulated.
